# Evaluating the Implications of a Coronary Artery Calcium Score of Zero (CAC = 0) in Modern Risk Prediction: Is Zero Truly Zero?

**DOI:** 10.7759/cureus.88513

**Published:** 2025-07-22

**Authors:** Samuel I Dos Santos Pereira, Adwaith Venugopal, Gevorg Manoukian, Hammad Buksh Ilahi, Mohd Hamza Masood, Mohamed Salaheddin Abdulrahman Shembesh, Anisa Anan, Nabeel Sufwan, Arham Sandeep Jain, Danielle L Dsouza, Ramsha Ali

**Affiliations:** 1 Internal Medicine, University of Buenos Aires, Buenos Aires, ARG; 2 Medicine, University of Central Lancashire, Preston, GBR; 3 Medicine, St. George's University School of Medicine, St. George's, GRD; 4 Cardiac/Thoracic/Vascular Surgery, Ziauddin University, Karachi, PAK; 5 Internal Medicine, International Higher School of Medicine, Bishkek, KGZ; 6 Medicine, University of Debrecen, Debrecen, HUN; 7 Medicine, Chattogram Maa-O-Shishu Hospital and Medical College, Chattogram, BGD; 8 Medicine, Jagadguru Sri Shivarathreeshwara (JSS) Medical College and Hospital, Mysore, IND; 9 Internal Medicine, All India Institute of Medical Sciences (AIIMS) Kalyani, Kalyani, IND; 10 Emergency Medicine, University Hospitals of North Midlands, Stoke-on-Trent, GBR; 11 Medicine and Surgery, Peoples University of Medical and Health Sciences, Nawabshah, PAK

**Keywords:** artificial intelligence (ai), atherosclerotic cardiovascular disease (ascvd), coronary artery calcium score (cacs), coronary artery disease (cad), major adverse cardiovascular events (mace), non-contrast computed tomography (ct)

## Abstract

Coronary artery calcium (CAC) scoring is a well-validated screening tool used to calculate the amount of calcified plaque deposited in coronary arteries from a computed tomography (CT) scan. It stratifies patients by risk to predict their future probability of cardiovascular disease and helps establish the ideal preventive therapies. Considering these factors, the purpose of this narrative review is to evaluate the latest research on the effectiveness of CAC scoring, explore the various limitations and challenges faced in utilizing this tool, and discuss alternative investigations commonly used to supplement it for risk stratification. To achieve this, a narrative review was conducted by searching recent literature through databases such as PubMed, Cochrane, and Google Scholar. Identified literature included large population cohort studies and systematic reviews from the last five years, focusing on CAC scoring, cardiovascular risk prediction, ethnicity, artificial intelligence (AI) integration, and secondary prevention. The literature identified generally shows that the validity of CAC scoring is strongly debated due to its variable efficacy in symptomatic versus asymptomatic patients and in the context of ethnic variations, with many studies having supported the validity of this scoring tool, but others challenging its ability to prognosticate cardiovascular disease due to the presence of these external factors, which could lead to an inaccurate representation of the score. As a result, a major recommendation has been to combine the calculated score with pre-existing patient risk factors when making clinical judgments to guide prompt, individualized primary and secondary preventive care. Studies have shown that patients with varying ethnic backgrounds and also those who are symptomatic for stable cardiovascular disease have confounding risk factors that can lead to a false representation of their score and could potentially be at a higher than predicted risk of major cardiovascular events even with a score of zero. In conclusion, the use of CAC scoring remains a valuable prognostic tool for predicting a patient’s cardiovascular prognosis; however, its interpretation must consider correlation with clinical, biochemical, and demographic contexts to optimize decision-making. The literature has also identified the potential for improving the precision and effectiveness of major adverse cardiovascular event (MACE) prediction using traditional scoring methods by incorporating AI, including automated scoring tools and calcium-omics models, into CAC scoring.

## Introduction and background

Coronary artery disease (CAD) is the most significant global cause of illness and death, accounts for more than 70% of sudden cardiac deaths, and contributes significantly to disability-adjusted life years (DALYs) worldwide [1,2]. Atheromatous blockage of coronary vessels, which precedes acute coronary events, as evidenced by plaque ruptures and the formation of thrombi, is the root cause of CAD [1]. Cognizant of this, the critical step of identifying asymptomatic people at risk for CAD becomes vital when creating decision-making guidelines for primary prevention [1]. One highly definitive indicator of atherosclerosis that provides predictive value in the pre-determination of cardiovascular risk stems from a specific marker: coronary artery calcium (CAC) [1,3]. One important detection method that aids in the identification of CAC is the use of cardiac computed tomography (CT), which is then quantified using the Agatston method. This method scores coronary plaques based on area and peak radiographic density [1,2,4]. The use of non-contrast CT to quantify CAC has gained prominence as a method to improve risk stratification and reclassification among those without symptoms [3,5]. While increased cardiovascular risk is associated with high calcium scoring markers, a non-detectable CAC (CAC = 0) is associated with a favorable risk profile, conveying a more advantageous prognosis [3,6].

Risk scores, which are determined through typical cardiovascular risk factors, such as pooled cohort equations, may result in the overestimation or underestimation of atherosclerotic cardiovascular disease (ASCVD) risk, partly due to outdated cohorts and a lack of individual-level risk calibration in diverse populations [7,8]. For those patients at intermediate risk (5%-20%), the ideal management of the diagnosis is often ambiguous. Traditional risk factor tools offer lower predictive value compared to the CAC score, which is a highly predictive tool of coronary heart disease (CHD), CVD, and mortality risk, as well as for improving risk discrimination and stratification [7]. Unlike risk calculators, CAC provides direct visualization of subclinical atherosclerosis, enabling superior risk discrimination [7]. Cardiovascular risk assessment is enhanced by the use of CAC scores in combination with traditional algorithms and guidelines, thereby benefiting clinical decision-making through more valuable information [9].

Guidelines such as the American College of Cardiology/American Heart Association (ACC/AHA), the Cardiac Society of Australia and New Zealand (CSANZ), the European Society of Cardiology/European Atherosclerosis Society (ESC/EAS), and the Canadian Cardiovascular Society (CCS) promote CAC scoring and aid in pharmacotherapy in patients between the ages of 40 and 75, asymptomatic patients, and/or if the calculated risk is determined to be intermediate or unreliable [10]. CACs have the capability to recategorize patients within the intermediate-risk population into either a lower- or higher-risk population, which exemplifies the immense benefit of CACs. For patients over 75, global parameters suggest the usefulness of CAC in reclassifying CV risk and predicting mortality. Guidelines also emphasize CAC scoring in asymptomatic individuals, especially those at intermediate risk, to inform statin therapy decisions [8,10]. Within the population of symptomatic patients, particularly those experiencing acute chest pain, a coronary artery calcium score (CACS) is not well delineated because it fails to detect non-calcified plaques that may still cause acute events [6,11].

General practitioners face a challenge when pinpointing and ruling out CAD in patients who present with atypical angina pectoris and nonspecific thoracic complaints. However, certain studies have indicated that CT-based CACS may offer diagnostic and exclusionary value for CAD in outpatient settings, distinguishing it from the CT CACS test typically used in primary care settings [11]. Outpatient settings often involve higher-risk patients and may utilize CACS to rapidly triage suspected CAD, whereas primary care application is typically more conservative and limited by patient selection [11]. While current practicality for CAC testing within the asymptomatic population experiencing moderate risk factors of CAD is established, CAC stand-alone test data is limited as pertaining to symptomatic patients experiencing acute coronary syndrome (ACS) as a leading differential diagnosis [12]. The rate of obstructive CAD (<1%) as well as long-term prognosis is favorable in CAC scores equal to 0 [13]. However, ACS cannot be ruled out when CAC = 0, which is prevalent in 1%-3% of those experiencing noncalcified plaque due to soft plaques or non-calcified lesions that evade detection by calcium scoring [13,14]. Performing CAC testing in advance of coronary computed tomography angiography (CTA) may be helpful in estimating coronary plaque burden and in guiding adjustments to the CTA protocol when heavy calcification is detected [13].

**Figure 1:**
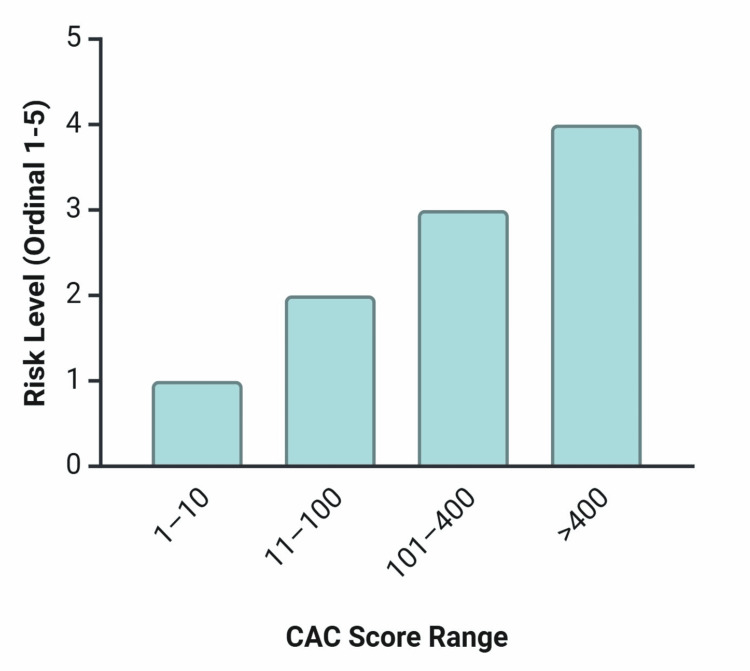
Interpretation of coronary artery calcium (CAC) score by ordinal risk level. The data used to create the figure was adopted from [18]. Image credits: Samuel Pereira

Within sensitivity, specificity, positive predictive value (PPV), and negative predictive value (NPV) parameters, identification rates of obstructive CAD in patients with a CAC score of zero were 92%, 54%, 31%, and 97% for stable CP compared to 90%, 67%, 32%, and 98% for acute CP, respectively [15]. Annual frequency of major adverse cardiovascular event (MACE) reports in patients with CAC = 0 was 0.5% (vs. 2.2%) for stable CP and 0.8% (vs. 8.5%) for acute CP [15]. Further study is warranted to determine the safety and efficacy of outcomes of CAC-only testing [15]. Notably, 16% of stable and 13% of acute CP patients with CAC = 0 were found to have obstructive or nonobstructive CAD, suggesting that relying solely on CAC may overlook cases that could benefit from management changes [15]. This finding emphasizes the risk of false reassurance in high-risk patients and supports the need for additional imaging or follow-up testing [15]. For those at greater risk, the presence of any calcium in the coronary arteries (i.e., CAC ≥ 1) is predictive of increased risk [16]. This is relevant, as a calcium score residing at the extreme ends of the continuum (scores of zero and ≥1000) has significant predictive value in identifying risk, as opposed to precise scores within the range of 1-1000, which offer minimal additional value with respect to risk stratification [17]. Intermediate scores are difficult to interpret and lack consistent thresholds for intervention across populations [17]. A simple interpretation of the CAC score associates a value of 0 with very low risk, while scores above 400 indicate a high likelihood of obstructive coronary disease [18]. An ordinal classification of CAC score ranges (Agatston units) and their associated probability of significant CAD is represented in Figure 1 [18]. Each range reflects a progressive increase in atherosclerotic burden, stratified into levels of clinical risk: 1-10: low probability; 11-100: mild or minimal coronary artery stenosis; 101-400: nonobstructive CAD likely, obstruction possible; >400: high likelihood of at least one significant coronary obstruction. These ordinal risk categories support treatment decisions, particularly regarding the initiation and intensity of lipid-lowering therapy [18].

One compelling subclinical marker of ASCVD risk, both in absolute and relative terms, is CAC, as measured by non-contrast cardiac CT, which has provided additional incremental prognostic data to estimates of risks originating from traditional risk factors [19]. Furthermore, prior analyses suggest that CAC testing can promote the personalized allocation of other therapies geared toward prevention (e.g., aspirin or statin) by pinpointing those with an improbability of receiving benefit, in addition to those who may have a greater probability of receiving benefits due to a higher absolute risk [20]. Both the American Diabetes Association and the ACC recommend CACS as a key strategy in supporting heart disease prevention efforts in diabetic populations. Guidelines suggest initiating statins for those with CAC values above the 75th percentile, while CAC screening itself is considered a class 2a indication. This method shifts treatment decisions toward personalized risk profiles, rather than relying solely on diabetes status. In cases of high CAC, additional evaluation with functional stress testing may further refine risk assessment [20].

This narrative review focuses exclusively on qualitative analysis, as is appropriate for this type of research. Studies were selected based on relevance to the topic and adherence to specific inclusion criteria. Only articles published within the last 10 years were considered, with the exception of two to three landmark studies deemed essential for contextual understanding. Furthermore, only human studies involving adult populations (aged 18 years and older) were included to ensure clinical relevance. The selection process prioritized peer-reviewed literature to maintain scientific rigor and credibility.

## Review

Use of CAC score in symptomatic vs. asymptomatic individuals

The effectiveness of CACS has been a commonly debated topic in predicting the risk of MACE for individuals who are asymptomatic vs. symptomatic of CHD, e.g., patients with stable angina. The CAC has been frequently shown in literature to have a strong prognostic value in asymptomatic people. A notable example is a systematic review, which has demonstrated strong evidence from multiple recent studies indicating that CACS is a highly promising and reliable predictor of MACE in asymptomatic individuals. Furthermore, a CAC score of 0 strongly suggests an extremely low probability of future MACEs, also known as a high NPV [21]. In fact, the implementation of CACS into clinical guidelines aims to screen high-risk asymptomatic patients for CHD, guiding risk stratification and the early commencement of preventive medical therapies against MACE, such as statin intensification [22].

**Figure 2:**
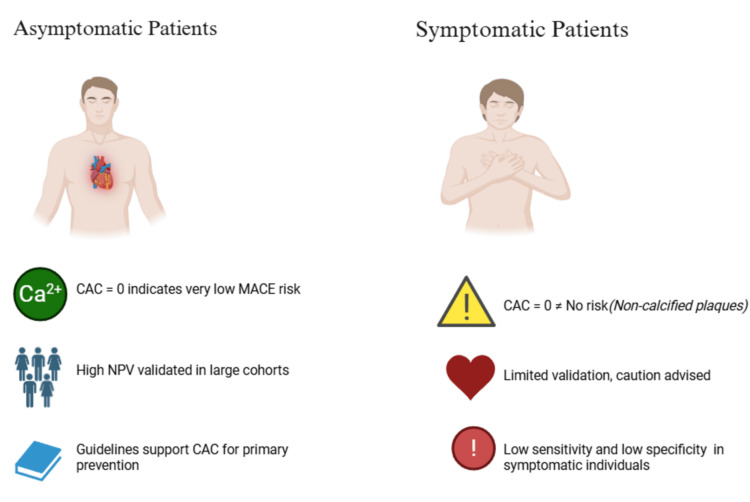
The difference in predictive value of a coronary artery calcium (CAC) score of 0 between asymptomatic and symptomatic individuals. Data adopted from [3,21,23,24]. Image credits: Samuel Pereira

Nevertheless, the validity of CAC scoring in symptomatic patients has been widely debated. The PROMISE trial, undertaken in 2017, investigated approximately 10,000 symptomatic patients suspicious for stable CHD and found that those with a CAC score of 0 were associated with a lower probability of plaque formation, a lower risk of MACE, and a reduced frequency of future revascularization interventions. This concept is described as the "Power of Zero," where CAC scores of 0 are closely associated with a lower ASCVD risk, while having a proportionately higher risk of cardiovascular mortality with increasing scores above 0 [23]. However, it has been frequently challenged for its weaker efficacy in specific patient groups. This was highlighted in a retrospective evaluation of 5144 symptomatic participants with a CAC score of 0, which found that 81 (15.7%) had evidence of atherosclerotic plaque on a coronary CTA [24]. This raises the question of how high-risk obstructive CHDs get missed despite a CAC of 0. This could either be explained by any coexisting metabolic conditions like uremia, which impact calcification in arteries through impeded bone mineral metabolism and subsequent deposition in arterial walls, or even the presence of non-calcified plaques, which can lead to CHD as well as these symptomatic features but are not clearly distinguishable on non-contrast CT scans due to them having similar densities as surrounding artifacts, including blood and epicardial fat [3]. Therefore, solely depending on CAC scores in symptomatic patients instead of clinical features could lead to underestimating the risk of MACE and missing out on potentially commencing early, life-saving treatment. Figure 2 visually illustrates the difference in predictive value of a CAC score of 0 between asymptomatic and symptomatic individuals.

Consequently, clinical practice has cautioned that CAC should not be used as a sole indicator of predicting the future risk of MACE and that a score of 0 does not automatically indicate low risk, a theory commonly termed the "Zero Calcium Paradox." Clinicians are also recommended to consider patients' clinical status and cardiovascular risk factors like age, smoking, and history of hypertension and diabetes when assessing this risk and initiating preventative measures [24].

Ethnicity and its effect on the reliability of the CAC score

**Figure 3:**
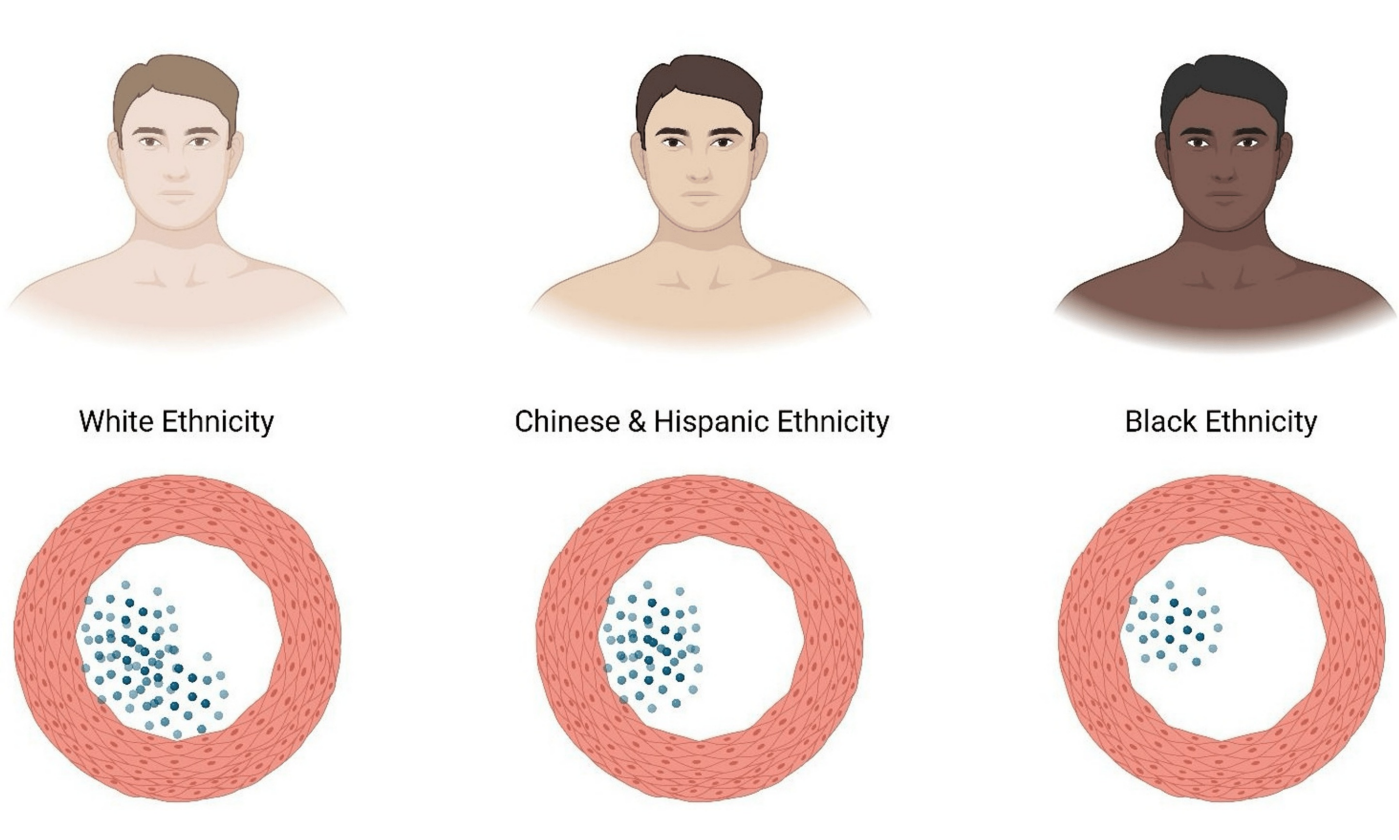
A pictorial representation of coronary artery calcium (CAC) prevalence in multi-ethnic groups. The White ethnic group exhibits the highest prevalence, the Black ethnic group the lowest, with the Chinese and Hispanic ethnic groups positioned in the middle. Data adopted from [25]. Image credits: Anisa Anan

CACS remains a validated tool for detecting atherosclerosis and estimating future cardiovascular risk. However, multi-ethnic data have revealed marked variations in CAC prevalence and risk detection across populations. Data from the Multi-Ethnic Study of Atherosclerosis (MESA) reported that CAC prevalence was highest among White populations, lowest among Black populations, and intermediate for Chinese and Hispanic populations, as illustrated in Figure 3 of the published data [25]. These patterns remained static even after adjusting for known risk factors, which suggested that ethnicity independently influenced coronary calcification [25].

Despite these differences in CAC burden, its predictive value remains uniform across different populations. CAC scores were significantly linked with incident CHD events across ethnicities [26]. Regardless of ethnic background, there is a strong association with higher CAC scores and all-cause mortality [27]. Both these studies confirmed that CAC is a valuable prognostic marker in diverse populations, although the risk varies.

Further reviewed ethnic backgrounds in CAC by analyzing coronary atherosclerosis across all four main ethnic groups. They reported significant differences in the presence and extent of CAC, highlighting that certain populations have a risk of CAD without pathways involving calcification. This finding shows that risk is lower in some ethnic groups, particularly Black individuals, who tend to have a CAC burden despite being associated with more risk factors [28].

Supporting this, Hispanic and non-Hispanic White patients undergoing percutaneous coronary interventions. It showed that the cohort of Hispanic patients had significantly low CAC scores despite having a higher number of risk factors [17]. This further strengthens the idea that standard CAC thresholds may underestimate the risk in some ethnic groups.

Collectively, these findings show the importance of contextualizing CACS within a patient's ethnic background. While the CAC is an effective tool for assessing cardiovascular risk, its interpretation should be enhanced by incorporating demographic and ethnic factors to improve accuracy and support more individualized clinical decision-making. Its interpretation should incorporate demographic and ethnic factors to enhance accuracy and support more individualized clinical decision-making. Beyond these demographic conditions, it has been observed that comorbid conditions such as chronic kidney disease, diabetes mellitus, and metabolic syndrome also significantly affect CAC and its interpretation. In patients with diabetes or metabolic syndrome, CAC tends to progress more rapidly and is linked to more cardiovascular events even when the CAC score is at a lower threshold, which highlights the aggressive nature of subclinical atherosclerosis in these populations [19]. Additionally, in individuals with chronic kidney disease, extensive vascular calcification may occur through non-atherosclerotic pathways, such as medial arterial calcification, which does not reliably reflect plaque burden. This can lead to overestimation of atherosclerotic risk when CAC is interpreted in isolation [20].

Artificial intelligence (AI)-enhanced imaging and machine learning (ML)

The integration of AI in CACS helps improve accuracy and precise diagnostics. The deep learning model's AI performance showed remarkable agreement with that of expert radiologists, as indicated by an ICC of 0.951 (95% CI: 0.933-0.964). The scoring process was significantly faster; the study utilized routine ECG-gated non-contrast cardiac CT scans with an "AI median score of 15 ± 2 seconds compared to 45 ± 24 seconds" for manual scoring [29]. These results demonstrate the clinical application of AI CACS for rapid and accurate risk stratification of patients and its potential to enhance cardiovascular evaluation of patients at risk for MACE [29].

AI presents a significant opportunity for timely intervention in cardiovascular health by facilitating the early identification of atherosclerosis before the manifestation of clinical symptoms. AI in the use of CACS has been shown to enhance the implementation of preventive therapies. Empirical evidence suggests that the detection of calcified coronary plaques is positively correlated with the initiation and adherence to both pharmacological interventions, such as statin therapy, and lifestyle modifications, including dietary changes, which collectively contribute to improved patient outcomes [30].

Furthermore, AI models have the ability to detect individuals at higher risk patients who may present with low CAC scores and are still vulnerable to MACE. This feature addresses a significant limitation in conventional CACS, which can misclassify individuals with low CAC scores as being at low risk. Although the application of AI for CACS and the prediction of MACE is promising, some challenges remain. Specific challenges include the need for large, diverse datasets to adequately train AI models, the risk of algorithmic bias, and the use of AI tools within existing clinical workflows [31].

These unresolved questions will require further exploration and are critical to the validation of AI models in the real world. Furthermore, the evolution, advancement, and deployment of various AI technologies may further improve cardiovascular risk stratification and prevention strategies when applied in conjunction with AI-derived CAC scores [31].

Integrating other scoring systems/biomarkers with the CAC score to predict MACE in both symptomatic and asymptomatic individuals

**Figure 4:**
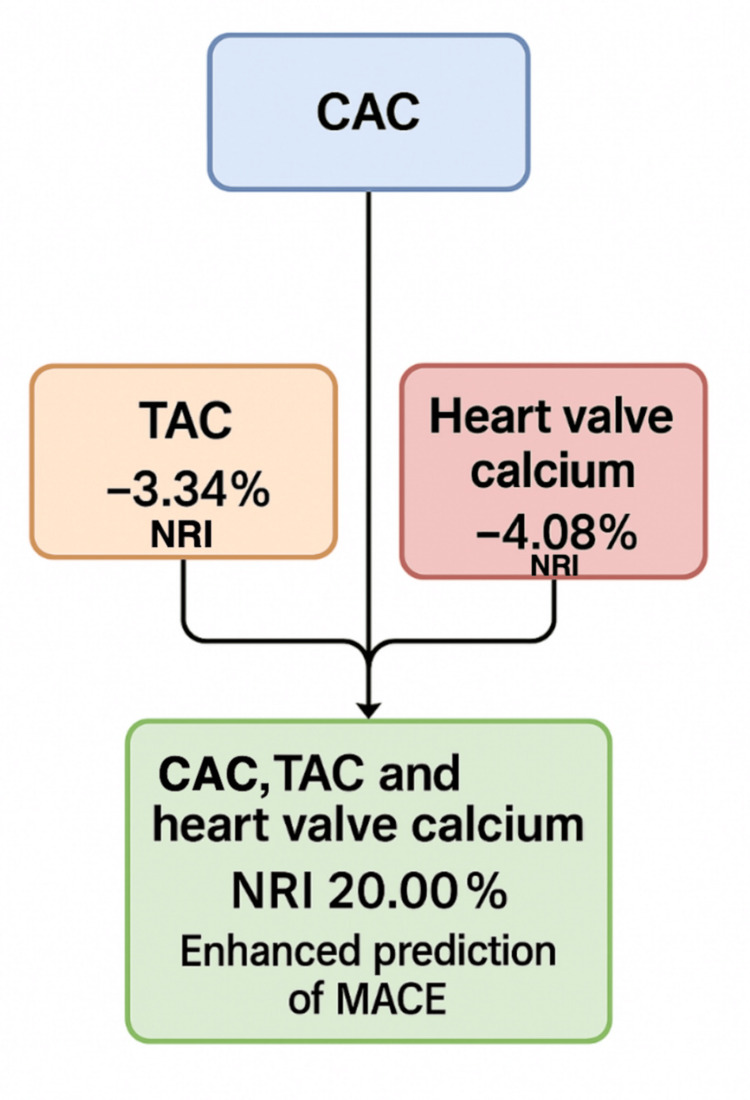
Enhanced major adverse cardiovascular events (MACE) risk prediction through combined calcium scoring. Data adopted from [32]. Image credits: Gevorg Manoukian

In addition to CACS as a marker of atherosclerotic burden and cardiovascular risk, emerging evidence continues to support the complementary value of the other biomarkers as add-on risk predictors alongside CACS. In the prospective cohort analysis derived from the UCC-SMART study, the separate inclusion of TAC (thoracic aortic calcium) or heart valve calcium scores did not significantly improve the predictive accuracy for MACE. TAC had an NRI (net reclassification improvement) of -3.34% (95% CI: -9.97 to 3.95) and heart valve calcium had an NRI of -4.08% (95% CI: -12.35 to 3.39), indicating only modest incremental prognostic information when considered separately. However, van't Klooster et al. systematically assessed that "adding TAC and heart valve calcium to CAC (Model V) resulted in an NRI that showed improvement in risk classification NRI 20.00% (95% CI 5.59-34.92) for the 10-year risk of MACE," visually illustrated in Figure 4 based on the published data [32]. The prognostic value of including all three calcium scores in a multivariate model with CAC (Model V) enhanced the prediction of MACE [32].

For their prospective cohort study, the researchers performed an analysis of the additive value of high-sensitivity troponin I (hs-TnI) and CAC scores for detecting patients at risk for MACE [33]. Combining these two diagnostic methods may help in identifying individuals at risk of having coronary artery blockages. In addition, adding hs-cTn and IL-6 to CACS may provide a more complete picture of cardiovascular risk stratification, especially among patients at high risk for MACE. In the PROMISE trial, those with "hs-cTn levels above the median (1.5 ng/L) had 2.1 times higher risk of MACE in unadjusted analysis (HR: 2.1; 95% CI: 1.3-3.6; P = 0.006)" [34]. IL-6 levels exceeding the average (1.8 ng/L) remain significantly correlated with MACE even after controlling for "ASCVD and high-risk plaque (HR: 1.9; 95% CI: 1.1-3.3; P = 0.03), CAC score (HR: 1.9; 95% CI: 1.0-3.4; P = 0.04), and SIS (HR: 1.8; 95% CI: 1.0-3.2; P = 0.04)" [34].

These findings highlight the necessity of expanding traditional assessment based solely on coronary calcification and advocate the adoption of different biomarkers into clinical cardiovascular risk evaluation paradigms, especially in patients with established or suspected atherosclerotic disease.

Applying the CAC score in younger adults vs. older people

Age itself, being an independent risk factor for CVD, is heavily weighted in all traditional risk assessment models; however, the weight of chronological age may possibly provide false reassurance to younger individuals, neglecting other important determinants, including lifestyle and psychosocial factors [35]. This echoes with evidence from the analysis demonstrating that the incidence of acute MI admission rates in the young to middle-age groups (>30 to <55 years) has not significantly declined compared to the general population [9]. Considering this, several studies, including the CARDIA study, have established a clear link between premature cardiac events in adulthood and CAC scores in young adults with ASCVD RFs [36].

Research from the MESA (2005) on individuals free of traditional ASCVD risk factors suggests that CAC increases steadily with age, although it is heavily affected by sex and ethnicity. In this study, more than one-third of young adults (predominantly of White ethnicity) with their predicted 10-year ASCVD risk being low had early manifestations of subclinical coronary atherosclerosis. The data helped calculate the CAC > 0 offset period to determine the recommended age for an index scan, aiding in primary prevention based on risk factors [25]. Studies have shown that using age as a sole determinant in deciding the need for CAC screening provides limited prognostic value, as it has been shown that younger adults with established cardiovascular risk factors like dyslipidemia, hypertension, and diabetes have been linked to prematurely having CAC scores over 0 [37].

To bridge the gap with young people, conceptualization of coronary age has recently emerged, incorporating traditional risk factors with CAC. This has served as an effective communication tool by determining the equivalent age at which a healthy subject may develop cardiovascular risk, similar to that of the patient. While the risk prediction is identical to the MESA CHD risk score, this provides a more relatable comparison for users than predicting risk solely based on chronological age [38].

Additional studies have verified the reliability of CAC scores both independently and in combination with diagnostic CCTA, as seen in the CONFIRM study on asymptomatic adults. The results showed that reclassification based on the severity of CAD by CCTA had incremental prognostic value over CACS in elderly patients (especially those with high-intermediate FRS), without added benefit in younger and middle-aged adults [39]. In contrast, another study found that the discriminative use of a CAC score = 0 in excluding obstructive CAD was age-dependent and less reliable in younger patients. A non-negligible proportion of obstructive CAD occurred among those without CAC in symptomatic patients < 60 years, which may put them at risk of MACE [40]. Based on the studies, a comparative analysis has been done as outlined in Table 1.

**Table 1:** Comparison of coronary artery calcium (CAC) scoring and cardiovascular risk assessment between younger adults and older individuals. Data adopted from [4,6,18,19,22,23,31,35]. Table credits: Samuel Pereira

Feature	Adult	Elderly	Evidence/Reference
Risk Assessment	Chronological age can be misleading; lifestyle and psychosocial factors are major.	Age is strongly weighted; traditional models potentially overestimate risk based solely on age.	[19]
MI Trends	No significant observation in admission rates for 30–55 years.	Improvement in MI incidence is noticeable.	[22]
CAC (Coronary Artery Calcium) in Primary Prevention	CAC in adults, including risk factors, predicts premature cardiac events.	CAC gradually increases with age, induced by sex and ethnicity.	[4,31]
Subclinical Atherosclerosis	One-third of adults (mostly White) with low 10-year ASCVD risk had early signs of disease.	Baseline CAC is proportional to age.	[18]
Concept of Coronary Age	Emerging as a better indicator of risk in young people than sequential age itself.	Useful across age groups; provides a relatable standard.	[6]
CAC Score vs. CCTA	CCTA doesn't add prognostic value over the CAC score alone in young or middle-aged people.	CCTA provides incremental prognostic value over CAC, especially in high-intermediate risk elderly.	[23]
Reliability of CAC = 0	Less reliable in excluding CAD in symptomatic patients <60 years; some obstructive CAD still occurs.	More reliable in excluding significant CAD in the elderly.	[35]

Evaluating the calcium-omics approach for predicting MACE

CVD continues to be the primary cause of death worldwide, and CAD is responsible for a substantial proportion of the burden. A dominant biomarker for CAD is coronary artery calcification (CAC), which is a marker of atherosclerotic plaque burden and is typically measured in Agatston score units [37]. However, the Agatston score, which is the most commonly used CTAC-based method, suffers in prediction potential because it sums calcium density without regard to the intricacies of the pathophysiology of such calcium. A novel technique, known as "calcium-omics," has been developed to overcome these limitations. This strategy relies on AI and ML to incorporate multiple hand-crafted features that are more interpretable and can capture the complexity of atherosclerotic calcifications. This article critically reviews the calcium-omics approach for its predictive utility in predicting MACE using time-to-event models [41].

**Figure 5:**
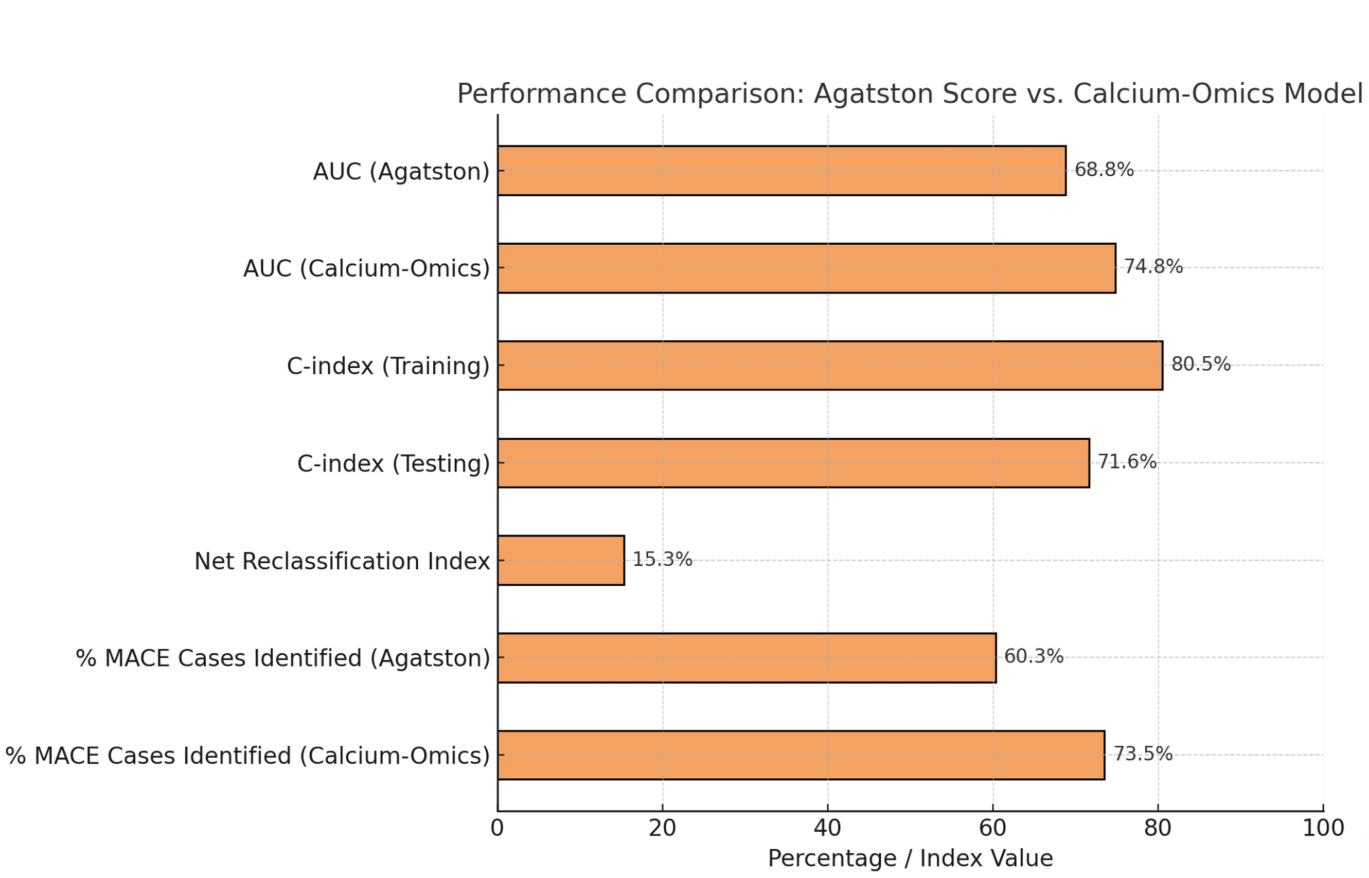
Performance comparison: Agatston score vs. calcium-omics model. Data adopted from [41]. Image credits: Gevorg Manoukian

A study by Hoori et al. utilized AI to analyze CT calcium score images of 2,457 patients, which included the full set of calcification features. The calcium-omics model performed better than the conventional Agatston score in predicting MACE [41]. In particular, the AUC for MACE prediction improved from 68.8% with the Agatston score to 74.8% with the calcium-omics model, representing a marked improvement in predictive performance [41]. Furthermore, the calcium-omics model achieved C-indexes of 80.5% in the training set and 71.6% in the testing set, which can robustly predict high- and low-risk patients. Assessment of the improvement in risk stratification revealed an NRI of 0.153, further confirming the model's ability to accurately reclassify patients into risk categories. Clinically, 73.5% of the high-risk group events were predicted by the calcium-omics model, with a 13.2% increase compared to the Agatston score, published data visually illustrated in Figure 5 [41]. The model has the potential to improve the early identification of patients who could benefit from intensified follow-up and targeted treatment.

The calcium-omics model represents an innovative step forward in cardiovascular risk stratification and, importantly, outperforms the classical Agatston score. Through the incorporation of high-dimensional, pathophysiologically meaningful features using AI and ML, calcium-omics enables a refined evaluation of the atherosclerotic load and an estimated future risk of MACE [41]. The observed enhancements in discrimination, reclassification, and clinical risk stratification position calcium-omics as a new approach in the era of "next-generation" tools that can be integrated into clinical workflows. Its implementation may lead to markedly improved early detection, better allocation of resources, and improved cardiovascular outcomes through more personalized and focused preventive approaches [42]. These results highlight the potential of calcium-omics to enhance the personalized prediction of cardiovascular risk; however, wider validation, along with integration into prospective clinical pathways, will be necessary for realizing the clinical potential of this technique.

Context

Even with modern medical advancements, CAD remains the leading cause of death worldwide. In over three decades, CVDs have remained the highest cause of mortality worldwide [43]. This statement highlights the significance of MACEs as a global public health concern. MACEs have a tremendous impact on morbidity, mortality, and healthcare costs. This global effect ensures that large amounts of research are being conducted to predict the likelihood of MACEs in both symptomatic and asymptomatic patients, guiding options for prevention and treatment and providing a true predictive value in symptomatic patients, especially those with other risk factors and metabolic comorbidities. Different ethnicities and demographics can affect the ability of CAC scores to predict MACEs. The integration of AI into CACS has shown promise in increasing the accuracy and efficiency of predicting MACE. CAC scores in combination with other biomarkers, such as TAC and heart valve calcium, showed a better predictive value for future MACE probability. While CAC has shown some predictive value in identifying MACEs in younger patients, it should be combined with other predictors to provide individualized care. Traditionally, CACS was used for preventive care; recent data support the notion that CAC scores can be helpful in the secondary prevention of MACE [44].

To conclude, while CACS provides important insights into predicting MACE, its interpretation should always be combined with clinical judgment, patient demographics, and other diagnostic modalities (such as blood tests, risk stratification scores, and radiology) to provide optimal prevention and treatment for patients. Due to this ongoing research, various tests are conducted to assess their contribution to predicting MACEs successfully. We aim to examine one such tool in depth as we assess the predictive value of CAC in identifying MACEs in patients presenting with or without typical symptoms. We reviewed freely accessible, peer-reviewed articles published in the English language. Our review examined the use of CAC as a standalone predictor and how its predictive value changes based on ethnicity and age. Furthermore, we looked at the potential benefits of combining CAC with other scoring systems and biomarkers, as well as integrating CAC with AI modules, to determine if these approaches would alter its predictive value in identifying MACEs in both symptomatic and asymptomatic patients.

Limitations

This narrative review has some limitations. First, most of the reviewed evidence is from observational cohort studies, and causality is always difficult to establish between polygenic risk scores (PRS), CAC burden, and MACE.

Second, while CAC is a well-established surrogate marker of subclinical atherosclerosis, it may not adequately reflect CAD risk in all populations. Importantly, Black individuals may have an increased cardiovascular burden despite resting or absent CAC, potentially related to non-calcified plaque or metabolic conditions such as uremia, which are not assessed on non-contrast CT.

Third, while AI/ML offers considerable potential to enhance CACS and MACE prediction, the clinical applicability of AI/ML is hampered by the necessity of large, ethnically diverse datasets and the enduring issue of algorithmic bias. Fourth, many studies lack the inclusion of elderly asymptomatic individuals, especially those aged 75 years or older, meaning that generalizability to this high-risk group is limited.

Lastly, even as enthusiasm for PRS-guided CAC screening has risen, its implementation in clinical practice will likely encounter significant barriers, including the incorporation into clinical workflows, provider education, patient acceptance, and ongoing reimbursement issues. In summary, these limitations highlight the necessity for inclusive, prospective validation studies prior to the widespread implementation of PRS-CAC strategies in the clinical arena.

## Conclusions

According to our review, CAC is a useful non-invasive tool for predicting the risk of MACE in asymptomatic patients at intermediate risk. It is highly reliable for these patients and can be of great clinical use in making decisions related to escalating preventive interventions. However, it is less reliable in symptomatic patients due to reduced sensitivity to non-calcified plaques and lower NPV. The CAC score has several limitations, including in patients with metabolic comorbidities, such as diabetes and chronic kidney disease, where vessel calcification can occur in the medial layer. Additionally, CAC is influenced by ethnicity; for example, Black individuals, compared to White and Hispanic individuals, tend to have less CAC burden but higher cardiovascular risk. Moreover, CAC is not reliable in younger patients (30-55), as a zero CAC score may not be accurate and should be correlated with biomarkers and/or diagnostic tests, such as CT angiograms. To counter these limitations, new AIs are being brought in to help interpret results faster and more accurately, but they remain a work in progress. Additionally, incorporating different scores, such as TAC and heart valve calcium, enhances risk prediction by capturing systemic calcific burden and extra-coronary vascular involvement. These markers contribute to prognostic information, especially in older adults or those with multivessel disease.

To conclude, while CAC scoring provides important insights into predicting MACE, its interpretation should always be combined with clinical judgment, patient demographics, and other diagnostic modalities (such as blood tests, risk stratification scores, and radiology) to provide optimal patient management. While AI shows great potential to improve the efficiency of using CAC, it requires further validation before it can be implemented everywhere.
